# Garlic allergy in an infant: Identification of alliin lyase 1 and 2 as causative allergens

**DOI:** 10.5415/apallergy.0000000000000201

**Published:** 2025-03-17

**Authors:** Yoshinori Morita, Karin Tsuchiya, Kazuyuki Sogawa, Naoki Shimojo

**Affiliations:** 1 Department of Pediatrics, IMS group, IMS Memorial Hospital, Tokyo, Japan; 2 Department of Biochemistry, School of Life and Environmental Science, Azabu University, Kanagawa, Japan; 3 Center for Preventive Medical Sciences, Chiba University, Chiba, Japan

**Keywords:** Alliin lyase, food hypersensitivity, garlic, infant

## Abstract

Garlic allergy is rare and is infrequently reported as a food allergy. This report details the case of a 13-month-old girl who developed an allergy to garlic. The consumption of a stew containing undercooked garlic triggered her allergic reaction. Blood tests revealed a garlic-specific IgE level of 10.3 kUA/L. In addition, in the skin prick test, a 4 × 4 mm, 2 × 2 mm, and 1 × 1 mm wheal was induced by raw garlic, garlic heated for 5 min, and garlic heated for 10 min, respectively. She had a known history of egg allergy. The foods consumed at the time did not contain eggs and included all other foods she had previously tolerated, except garlic. Therefore, we diagnosed her with a garlic allergy. We performed western blotting and mass spectrometric analysis and identified alliin lyase 1 and alliin lyase 2 as the major allergens. We further confirmed that the patient’s sera reacted with recombinant alliin lyase 1 and 2. To our knowledge, this is the first report to accurately demonstrate the involvement of alliin lyase 1 and alliin lyase 2 in a patient with garlic allergy. Moreover, it highlights the differences in reactivity to garlic heated for different durations. In patients with garlic allergy, reactivity to heating time should be examined with skin prick tests.

## 1. Introduction

In developed countries, 5% to 10% of infants have food allergies, with eggs and cow’s milk being the most common triggers [1]. Garlic, a member of the Amaryllidaceae family, is used widely as a seasoning and medicine. Although occupational garlic allergies due to inhalation or contact have been reported [2], food allergies to garlic are uncommon [3, 4]. Herein, we report a case of garlic allergy in an infant and the causative allergens identified after a series of tests.

## 2. Case report

A 13-month-old female ingested a stew mixed with a commercially available tube of raw garlic product that was heated in a microwave oven for 20 seconds. Thirty minutes later, she developed urticaria involving the neck, submandibular region, and upper anterior chest. Despite receiving an oral antihistamine (fexofenadine hydrochloride 15 mg), her symptoms did not improve, prompting a visit to the emergency department. Upon arrival, she presented with generalized urticaria, erythema, and worsening cutaneous symptoms that were refractory to prehospital treatment. Other systemic symptoms were absent, including hypotension, gastrointestinal symptoms, and respiratory distress. However, irritability was noted. Following a single intramuscular injection of adrenaline, her symptoms resolved rapidly with no recurrence, including urticaria. After a brief period of observation in the emergency department, she was discharged home. The infant had a history of urticaria from egg ingestion.

Serological test findings revealed a total IgE level of 166 IU/mL. Specific IgE levels for garlic, egg white, and ovomucoid, measured using the ImmunoCAP system, were 10.3, 3.61, and 3.53 kUA/L, respectively. We performed skin prick tests (SPTs) to evaluate hypersensitivity to garlic, the effect of heat on its allergenic properties, and potential cross-reactivity to other foods. Garlic was wrapped in aluminum foil to prevent burning and heated in a frying pan for a specified duration. In the positive control (histamine) test, a 5 × 5 mm wheal appeared, whereas in the negative control (saline) test, no wheal was observed. In the prick-to-prick test, a 4 × 4 mm, 2 × 2 mm, and 1 × 1 mm wheal was induced by raw garlic, garlic heated for 5 min, and garlic heated for 10 min, respectively. Wheals were not induced by raw or heated onions or leeks. The stew contained everything she had already eaten previously except garlic and no eggs. Although the patient had a known history of egg allergy, based on the clinical presentation and diagnostic findings, we diagnosed her with a garlic allergy.

**Figure 1.**
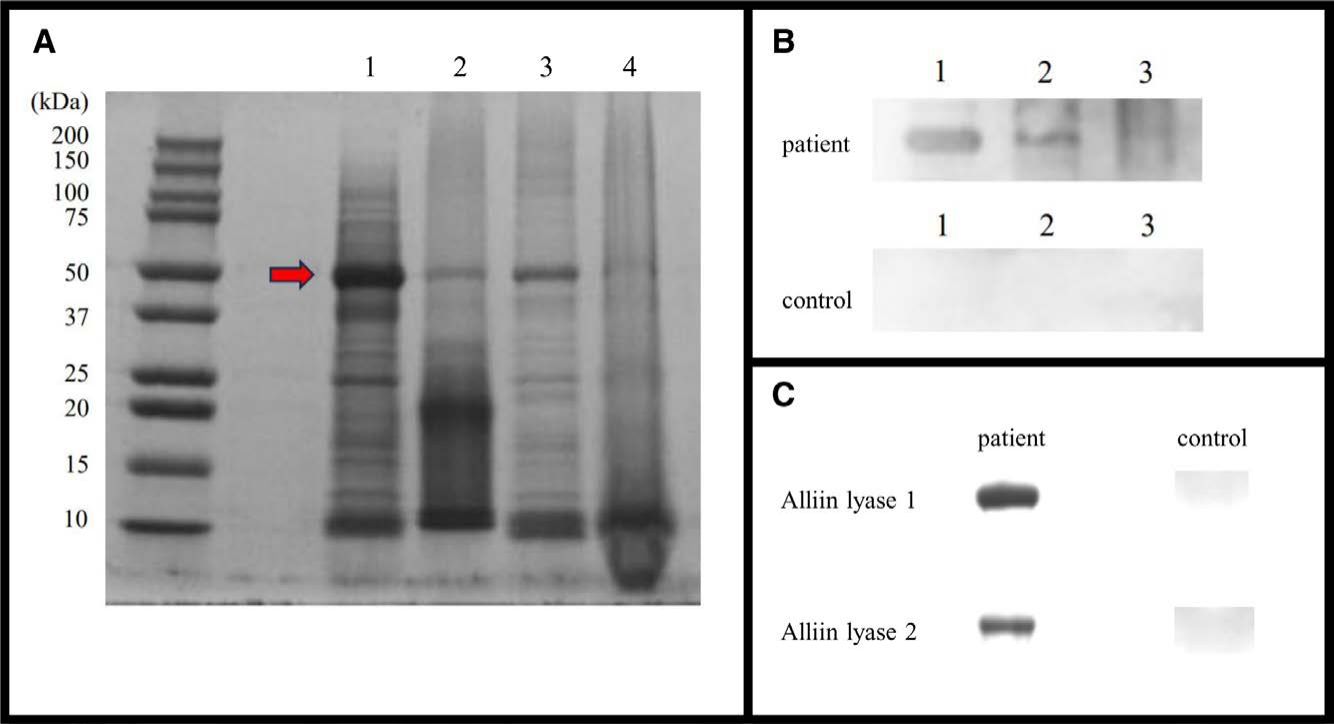
Western blot results. (A) SDS-PAGE. Lane 1: raw garlic (sliced), lane 2: raw grated garlic product, lane 3: 10-min heated garlic, lane 4: 15-min heated garlic. (B) IgE western blotting with serum from patient and healthy volunteers. Lane 1: raw garlic (sliced), lane 2: raw grated garlic product, lane 3: 15-min heated garlic. (C) Western blotting of alliin lyase 1 (upper panel) and alliin lyase 2 (lower panel). SDS-PAGE, Sodium dodecyl sulfate–polyacrylamide gel electrophoresis

We then performed an immunological analysis of crude garlic extracts, as previously described (Fig. 1A) [5]. Proteins were analyzed via western blotting for immunoreactivity. IgE bands were observed only in patient sera and disappeared in the garlic sample heated for 15 minutes (Fig. 1B). Proteins were identified using liquid chromatography-tandem mass spectrometry, with trypsin-digested peptides analyzed via a hybrid ion-trap Fourier transform mass spectrometer and matched to the UniProt database. The bands corresponded to alliin lyase 1 (Q01594) and alliin lyase 2 (Q41233), with sequence identities of 52.06% and 47.36%, respectively (Table 1). Reactivity was confirmed via western blotting using recombinant alliin lyase 1 and 2 expressed in *Escherichia coli*. Bands were observed only in patient sera, confirming these proteins as the major allergens in garlic for our patient (Fig. 1C). Detailed methods are provided in the Supplementary Material, http://links.lww.com/PA9/A62.

**Table 1.** Allergen identification test results

Accession	Protein Name	UProt E. Name	MW [kDa]	Calc.pI	Coverage
Q01594	Alliin lyase 1	(ALLN1_ALLSA)	55.6	8.12	52.06%
Q41233	Alliin lyase 2	(ALLN2_ALLSA)	54.1	7.37	47.36%

Tests indicated that garlic cooked for at least 15 minutes could be safely ingested, but the parents declined an oral food challenge, deeming garlic unnecessary in the infant’s diet.

Written consent was obtained from the parents, and the study was approved by the ethics committees of IMS Memorial Hospital (2022-07) and Azabu University (2004).

## 3. Discussion

Reports on garlic allergies are limited, and research on allergen identification is scarce. While alliin lyase is a known major allergen, this case is the first to identify alliin lyases 1 and 2 accurately. SPT and immunoblotting showed significantly reduced allergenicity with increasing heat. Therefore, differences in reactivity due to heating should be considered when evaluating garlic allergy.

Cross-reactivity within the Amaryllidaceae family is possible in garlic allergy. A Spanish study reported 27 cases of hypersensitivity to onions or garlic among 8109 patients, with 7 sensitized to both [6]. Similarly, a Saudi study found that 15 of 108 adults were sensitized to onion or garlic, with 12 testing positive for both [7]. These findings suggest significant cross-reactivity. However, in our patient, SPTs showed no cross-reactivity with onion or leek, emphasizing the need for individual assessment of cross-reactivity and tolerance in garlic allergy.

Garlic proteins containing alliin lyase are heat-labile [4, 8]. One study showed reduced allergenicity after 30 minutes of heating, with allergenic bands varying by heating duration [4]. In our case, the 50-kDa alliinase band was detected in raw garlic, raw grated garlic product, and garlic heated for 10 minutes, but it disappeared after 15 minutes. The faint band in the raw grated garlic product (Fig. 1A) may result from chemical additives, low protein concentration, or mechanical grating. Similarly, in Figure 1B, the raw grated garlic product is thinner than raw garlic, indicating differences in allergenicity based on product type and state. Heating for at least 15 minutes appears to reduce garlic allergenicity, which is relevant for food processing and allergen management. Patients with garlic allergy may tolerate long-cooked dishes but react to briefly cooked ones, such as stir-frying.

In this study, we used a protein expressed in *E. coli* to investigate the patient’s reactivity. Although *E. coli*-derived proteins lack post-translational modifications such as glycosylation, which may affect their structure and allergenicity [9], specific reactivity was observed only in this patient. Despite these limitations, this result supports the protein’s potential role as the allergen. Further investigation of other potential allergens is warranted.

While garlic allergy can manifest with various symptoms, patients with garlic allergy rarely present with food allergy or anaphylaxis. In our patient, generalized urticaria and worsening cutaneous symptoms refractory to prehospital treatment were observed, with rapid resolution following a single intramuscular injection of adrenaline. Alliin lyases 1 and 2 were identified as the causative allergens; these proteins should be further examined in individuals who experience severe allergic reactions to garlic ingestion.

## Conflicts of interest

The authors have no financial conflicts of interest.

## Author contributions

Morita managed the patient and drafted the manuscript. Sogawa performed the examinations, contributed to manuscript preparation, and analyzed the data. Tsuchiya performed the examinations. Shimojo provided therapeutic advice and critically reviewed the manuscript. All authors approved the final version.

## Editing support

We thank Editage (www.editage.com) for English language editing.

## Supplementary material

Supplementary Material can be found via 10.5415/apallergy.2022.12.e38

Supplementary Material

Click here to view
